# Effect of Superparamagnetic Iron Oxide Nanoparticles-Labeling on Mouse Embryonic Stem Cells

**DOI:** 10.22074/cellj.2016.3719

**Published:** 2015-07-11

**Authors:** Hamed Parsa, Karim Shamsasenjan, Aliakbar Movassaghpour, Parvin Akbarzadeh, Bahram Amoghli Tabrizi, Nima Dehdilani, Parisa Lotfinegad, Farzaneh Soleimanloo

**Affiliations:** 1 Hematology and Oncology Research Center, Tabriz University of Medical Sciences, Tabriz, Iran; 2 Blood Transfusion Research Center, High Institute for Research and Education in Transfusion Medicine, Tehran, Iran; 3 Department of Pharmaceutical Biotechnology, Faculty of Pharmacy, Tabriz University of Medical Sciences, Tabriz, Iran; 4 Department of Clinical Sciences, Faculty of Veterinary Medicine, Tabriz Branch, Islamic Azad University, Tabriz, Iran; 5 Department of Anesthesiology, Tabriz University of Medical Sciences, Tabriz, Iran

**Keywords:** Iron Oxide, Mouse Embryonic Stem Cells, Cell Tracking

## Abstract

**Objective:**

Superparamagnetic iron oxide nanoparticles (SPIONs) have been used to label mammalian cells and to monitor their fate *in vivo* using magnetic resonance imaging (MRI). However, the effectiveness of phenotype of labeled cells by SPIONs is still a matter of question. The aim of this study was to investigate the efficiency and biological effects of labeled mouse embryonic stem cells (mESCs) using ferumoxide- protamine sulfate complex.

**Materials and Methods:**

In an experimental study, undifferentiated mESCs, C571 line, a generous gift of Stem Cell Technology Company, were cultured on gelatin-coated flasks. The proliferation and viability of SPION-labeled cells were compared with control. ESCs and embryoid bodies (EBs) derived from differentiated hematopoietic stem cells (HSCs) were analyzed for stage-specific cell surface markers using fluorescence-activated cell sorting (FACS).

**Results:**

Our observations showed that SPIONs have no effect on the self-renewal ability of mESCs. Reverse microscopic observations and prussian blue staining revealed 100% of cells were labeled with iron particles. SPION-labeled mESCs did not significantly alter cell viability and proliferation activity. Furthermore, labeling did not alter expression of representative surface phenotypic markers such as stage-specific embryonic antigen 1 (SSEA1) and cluster of differentiation 117 (CD117) on undifferentiated ESC and CD34, CD38 on HSCs, as measured by flowcytometry.

**Conclusion:**

According to the results of the present study, SPIONs-labeling method as MRI agents in mESCs has no negative effects on growth, morphology, viability, proliferation and differentiation that can be monitored *in vivo*, noninvasively. Noninvasive cell tracking methods are considered as new perspectives in cell therapy for clinical use and as an easy method for evaluating the placement of stem cells after transplantation.

## Introduction

Mouse embryonic stem cells (mESCs) have the ability of self-renewal (1, 2). These properties lead to our understanding of ESCs in disease mechanisms, monitoring drug safety and effectiveness, and considering the human ESCs (hESCs) as a novel and unlimited source of cells for using in the therapy of serious diseases and damages (traumatogenic occlusion) caused by injuries (3, 4). However, in order to find practical application for cell therapy, it is necessary to develop suitable methods for cell fate tracking, cell migration and cell final destination in the body.

Previously *in vivo* cell monitoring using radionuclide labels such as indium-111 was common, but it was shown to have the potential toxicities to some cell types or some clinical manifestations (5, 6). An interest in using magnetic resonance imaging (MRI) to follow trafficking behavior of cells labeled with superparamagnetic iron oxide nanoparticles (SPIONs) is increasing. Such cell trafficking studies would be a promising method for evaluation of cell-based repair, replacement or treatment strategies (7, 8).

SPIONs detectable by MRI are used to investigate liver, spleen (9, 10), lymph nodes (11) and gastrointestinal tract pathologies (12). Sipe et al. (13) used this method for intracellular labeling of human mononuclear cells. SPION-labeled cells are also detectable by MRI *in vivo*, so labeling with this method may also be possible for ESCs.

The amount of iron oxide that would be required for clinical MRI is small in comparison with the physiological iron stores (14). Therefore, due to low toxicity of SPIONs, they may be easily used in diagnostic medical testing (15).

There are numeral reports regarding SPIONs to label mammalian cells in animal model and application of MRI in order to monitor their position or migration *in vivo* (16-19). There are also many reports regarding SPIONs in ESCs (20, 21). However, the effect of SPIONs on the qualities of ESCs is not still known. It is apparent that SPIONlabeled ESCs migrate in the tissue of the organism, differentiate and adopt new features that are mainly dependent on their position in the target tissue (20, 22). However, questions still remain on the effectiveness of magnetic labeling of ESCs as well as its effects on cell behavior, division and/or differentiation processes. Therefore, in our study, we tested the effect of two following commercially available Food and Drug Administration (FDA)- approved agents on growth and differentiation of mESCs *in vitro*: i. ferumoxides that is a suspension of dextran-coated SPION used as MRI contrast agent and ii. protamine sulfate that is used *ex vivo* as a cationic transfection agent.

## Materials and Methods

This experimental study was done using mESCs, C571 line, a generous gift of Stem Cell Technology Company, USA, after receiving Ethical Committee approval of Faculty of Veterinary Medicine, Tabriz Branch, Islamic Azad University, Tabriz, Iran.

### Optimization of iron content nanoparticles

In this research, Hela cells were used for cultivation and optimization dose of iron content nanoparticles for cell labeling. Thus Hela cells were cultured in six-well plates in RPMI-1640 (Gibco, USA) with 10% fetal bovine serum (FBS, Gibco, USA) and different concentrations of ferumoxide (ENDOREM, Guerbet, France, 25, 50 and 100 μg/ ml) and protamine-sulfate (Sigma, USA, 0.3, 3 and 30 μg/ml). Cells were incubated for 24 hours, and they were then washed with phosphate-buffered saline (PBS, Gibco, USA). Iron inside the cells was visualized by prussian blue staining.

### Mouse embryonic stem cells culture

Undifferentiated mESCs were cultured based on previously reported methods by Shen and Qu (23). Briefly, PBS with 1% gelatin was poured into 96- well culture plates and incubated for 30 minutes at room temperature. Excess gelatin was removed by aspiration and the cells were rinsed with PBS. mESCs were suspended at a density of 1-3×10^5^ in knockout Dulbecco’s modified Eagle medium (KO-DMEM, Gibco, USA) with 20% (v/v) heatinactivated FBS, 100 U/ml penicillin (Gibco, USA), 100 mg/ml streptomycin (Gibco, USA), 2 mM 2-mercaptoethanol (Sigma, USA), 0.1 mM nonessential amino acids (Sigma, USA), 2 mM L-glutamine (Sigma, USA) and 10 ng/ml murine recombinant leukemia inhibitory factor (GenScript USA, Inc., USA, mESC cell medium). Cells were incubated under conditions described above. The third passage of cells was used for all experiments.

After 2-3 days of cultivation, non-adherent cells were removed by aspiration and attached cells were washed twice with PBS. The culture medium was changed every 2 days.

### Labeling cells by superparamagnetic iron oxide nanoparticles

When the cells reached 70-80% confluency, they were used for labeling procedure with Endorem SPIONs (Guerbet, France)-protamine sulfate (Sigma, USA) complex. Protamine sulfate was prepared as a fresh stock solution of 1 mg/ml in distilled water at the time of use. To evaluate the effect of SPIONs on growth and viability of cells and to observe the optimization of SPIONs, we used Hela cells for labeling with different concentrations (25, 50 and 100 μg/ml of ferumoxides (ENDOREM, Guerbet, France) and 0.3, 3 and 30 μg/ml of protamine sulfate per culture medium) at first. The same optimization procedure was done on mESCs. Subsequently they were incubated with 100 μg/ml ferumoxide and 3 μg/ml protamine sulfate of culture medium at 37˚C with 5% CO_2_. After incubation, mESCs were washed with PBS to remove any SPIONs not in taken by the cells. Adherent cells were detached with trypsin- ethylenediaminetetraacetic acid (EDTA, Gibco, USA), counted with a haemocytometer, and used for further experiments.

### Prussian blue staining

For prussian blue staining of ferric iron, cells were fixed with methanol, washed with PBS, incubated for 30 minutes with 5% potassium ferrocyanide (Sigma, USA) in 6% hydrochloric acid (Merck, Germany), washed with PBS again and stained with Fuchsin (Merck, Germany).

### Formation of embryoid bodies (EBs) in vitro

The cells were grown on gelatin-coated tissue culture flasks and maintained in an undifferentiated state using KO-DMEM with 20% (v/v) heat-inactivated FBS, 100 U/ml penicillin, 100 mg/ml streptomycin, 2 mM 2-mrcaptoethanol, 0.1 mM nonessential amino acids, 2 mM L-glutamine and 10 ng/ml murine recombinant leukemia inhibitory factor for a week.

### Differentiation stage

To initiate mESCs differentiation into mouse hematopoieitc stem cells (mHSCs), we modified a protocol based on previously reported methods by Shen and Qu (23). Briefly mouse EBs (mEBs) were counted and reseeded in 12-well plates in KO-DMEM, (20% FBS) with hematopoietic cytokines cocktail including 20 ng/ml human stem cell factor (hSCF), 20 ng/ml interleukin 3 (IL3), 2 ng/ml IL6, 20 ng/ml human Fms-related tyrosine kinase 3 ligand (hFlt3-L), and 50 ng/ml human thrombopoietin (hTPO) (all from GenScriptUSA, Inc., USA). Media was changed every 2 days.

### Phenotypic evaluation of cells

The undifferentiated cells were washed with 1 ml of PBS and incubated for 30 minutes on ice in dark to bind to fluorescently labeled antibodies [stage-specific embryonic antigen 1 (SSEA1) and cluster of differentiation 117 (CD117)] (Santa Cruz Biotechnology, USA). After differentiation of EBs, differentiated cells were washed and incubated with specific antibodies (CD34 and CD133) for mouse cells, whereas isotype control cells were stained with isotype immunoglobulin G (IgG) fluorescein isothiocyanate (FITC)/ phycoerythrin (PE) antibodies from the same company. Specific antibodies that were used in experiments to analyze the expression of cell surface markers were as follows: PE conjugated anti-mouse SSEA-1 (Santa Cruz, USA), FITC conjugated anti-mouse CD117 (Biolegand, USA), PE conjugated anti-mouse CD34 (Santa Cruz, USA), FITC conjugated anti-mouse CD133 (Biolegand, USA) and FITC conjugated anti-mouse CD38 (Santa Cruz, USA). Fluorescence activated flowcytometry was performed using FACSCaliber flowcytometry (Becton and Dickinson, FranklinLakes, NJ, USA). Live cells used for the analysis were gated based on forward angle light scatter (FSC) and side angle light scatter (SSC) and further analyzed using the CellQuest Pro software (Becton and Dickinson, USA). As a control, cells were stained with only isotype monoclonal antibody in order to eliminate nonspecific background staining.

All of experiments were done triplicates and were repeated for five times.

### Data analysis

A t test was run between labeled and unlabeled cells in order to evaluate their conditions. The data were presented as the mean ± standard deviation (SD) and P<0.01 was considered significant.

## Results

### Optimization of ferumoxide-protamine sulfate for cell labeling

**Fig.1:**
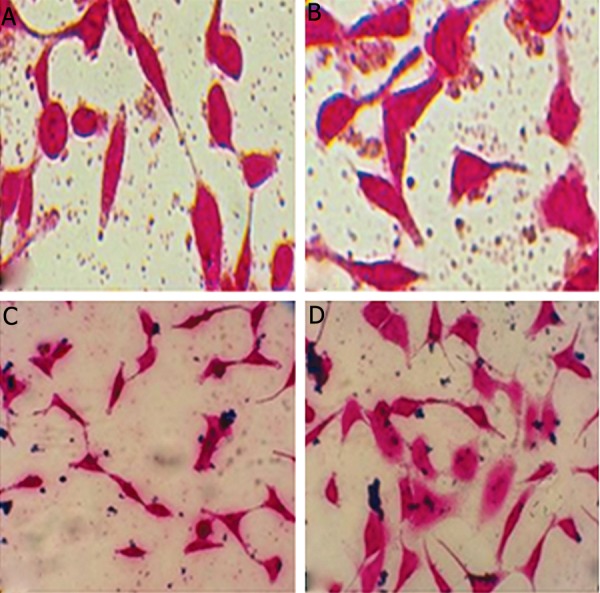
A, B. Ferumoxide uptake by Hela cells. Before labeling with ferumoxide nanoparticles, C and D. After labeling with ferumoxide nanoparticles (×40).

**Fig.2:**
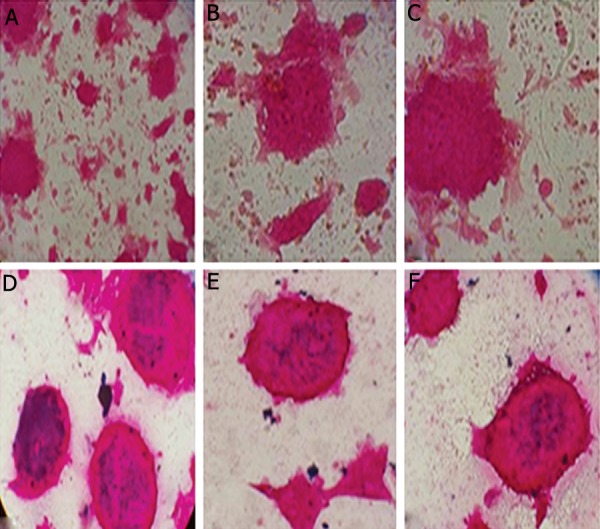
A, B, C. Ferumoxide uptake by mESCs. Before labeling with ferumoxide nanoparticles, D, E and F. After labeling with ferumoxide nanoparticles (×40). mESCs; Mouse embryonic stem cells.

Hela cells used for cultivation and optimization dose of iron content nanoparticles were cultured in six-well plates in RPMI-1640 with 10% FBS and different concentrations of ferumoxide (25, 50 and 100 μg/ml) and protamine-sulfate (0.3, 3 and 30 μg/ml). Cells were incubated for 24 hours, and they were then washed with PBS. Iron inside the cells was visualized by prussian blue staining. Cell counting of Prussian blue-stained cells in the suspension revealed that after the 24 hours, 100% of cells were labeled (Fig.1), and ferumoxide wasn’t toxic to cells. The same steps were repeated for labeling of mESCs with mentioned concentrations of ferumoxide-protamine sulfate complex. The results show that labeling of mESCs with 100 μg ferumoxide and 3 μg protamine sulfate per each culture media is the optimal dose for labeling of mESCs (Fig.2).

### Labeling of embryonic stem cells with optimal concentration of superparamagnetic iron oxide nanoparticles

Uptake of ferumoxides in the mESCs was revealed by prussian blue staining and stained cell percentage was determined by counting 1000 cells under a microscope. Stainable iron was not detected in the control ESCs (cells unlabeled with SPION). A high-efficiency labeling of SPION using ferumoxide-protamine sulfate with optimal dose of 100 μg/ml and 3 μg/ml was observed. After 24 hours, the presence of iron in 100% of ESCs was detected (Fig.2).

### Effect of superparamagnetic iron oxide nanoparticles on the self-renewal and viability of mouse embryonic stem cells

**Fig.3:**
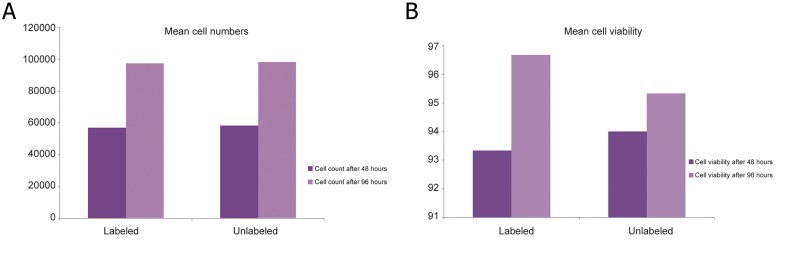
A. Comparison of cell number of labeled and unlabeled mESCs during 4 days with a 48-hour interval and one passage under standard conditions and B. Comparison of viability of labeled and unlabeled mESCs during 4 days with a 48-hour interval and one passage under standard conditions. mESCs; Mouse embryonic stem cells.

mESCs were collected after 48 and 96 hours. The proliferation rate of SPION-labeled cells was compared with control cells using microscopic cell count method. We detected no differences at the analyzed time intervals between control and SPION-labeled cells (P>0.05). At the same time, we stained collected mESCs with trypan blue (Merck, Germany). Viable mESCs were counted and percentage of viable cells was calculated. Our results showed nodifferences in viability between labeled and unlabeled cell groups (P>0.05, Fig.3).

### Effect of superparamagnetic iron oxide nanoparticles on the cell surface markers of mouse embryonic stem cells in comparison with control

**Fig.4:**
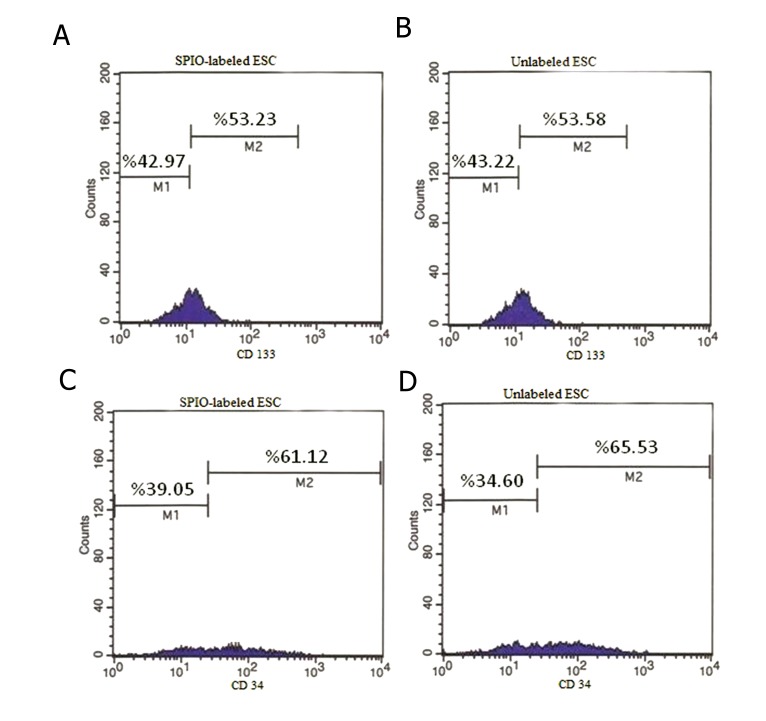
A, B. Analysis of the mESC surface markers in labeled and unlabeled status before differentiation. Flowcytometric analysis was performed for the specific markers of embryonic stem cells using SSEA1-PE, CD117-FITC, CD34-PE and CD38-FITC antibodies. Expression levels of SSEA-1 and CD117 in labeled and unlabeled mESC, C and D. Expression levels of CD34 and CD38 in labeled and unlabeled mESC. mESC; Mouse embryonic stem cells, SSEA1; Stage-specific embryonic antigen 1 conjugated, PE; Phycoerythrin, CD; Cluster of differentiation, FITC; Fluorescein isothiocyanate, SPIO; Superparamagnetic iron oxide, M1; Negative population and M2; Positive population.

mESCs were collected after 96 hours for flowcytometric analysis. Therefore, SSEA-1 and CD117 expression levels were analyzes to find out about the undifferentiated status of mESCs. The results revealed that mESCs in comparison with unlabeled cells didn’t change the population of surface marker of SSEA1 and CD117 expressing cells (P>0.05). Although almost all the SPION-labeled and unlabeled ESCs expressed the SSEA1 and CD117 surface markers, they showed no expression of hematopoietic surface markers, CD38 and CD34(Fig.4).

### Effect of superparamagnetic iron oxide nanoparticles on differentiation of mouse embryonic stem cells

**Fig.5:**
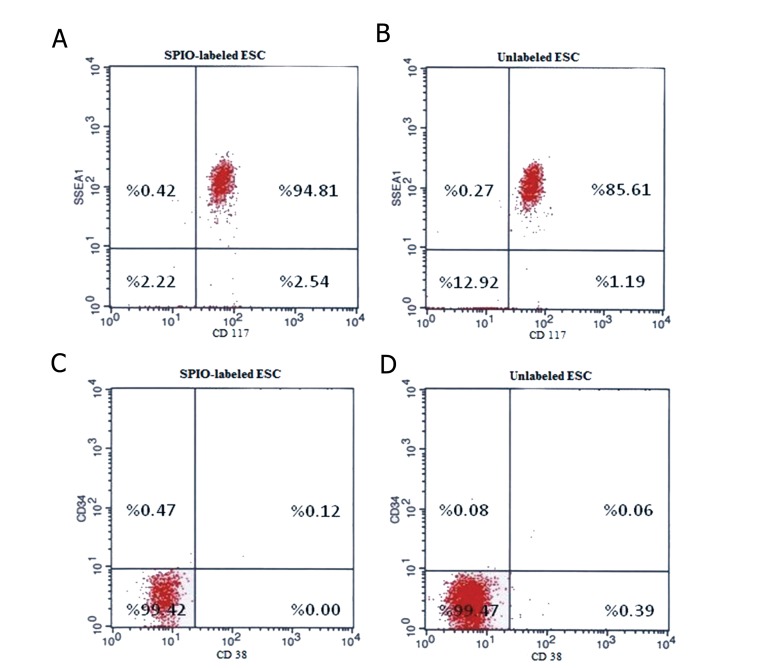
Analysis of the hematopoietic stem cells in labeled and unlabeled status after differentiation. Flowcytometric analysis was performed for the specific markers of hematopoietic stem cells using CD133-FITC and CD34-PE antibodies. A, B. Expression levels of CD133 in labeled and unlabeled status, C and D. Expression levels of CD34 in labeled and unlabeled status. CD; Cluster of differentiation, FITC; Fluorescein isothiocyanate, PE; Phycoerythrin, SPIO; Superparamagnetic iron oxide, ESC; Embryonic stem cell and SSEA1; Stage-specific embryonic antigen 1.

For hematopoietic differentiation, 8-day old EBs were transferred to gelatin-coated 24-well plates and maintained in iscove’s modified dulbecco medium (IMDM)-LIF^+^ (Gibco, USA) with 20% FBS for 2 days. After that EBs in 2 groups of SPIONs-labeled and unlabeled were seeded in 12-well plates for 7 days. The differentiation media used was IMDM with 20% FBS supplemented with cytokines including 20 ng/ml hSCF, 20 ng/ml human IL3 (hIL3), 2 ng/ml mIL6, 20 ng/ml hFlt3-L, and 50 ng/ml hTPO for hematopoietic differentiation. Hematopoietic cell surface markers expressing cell population were evaluated in both labeled and unlabeled cells. The results revealed that there was no difference in population of CD34 and CD133 expressing cells among two groups (Fig.5).

## Discussion

Progress in the field of cell therapy needs qualitative and quantitative evaluation of trafficking mechanisms in target tissue, homingway, proliferation and differentiation of studied cells. The advanced MRI technology has made it possible to detect engrafted cells *in vivo*, noninvasively; therefore, it’s necessary to label cells with contrast agents. SPIONs are considered as the prevailing contrast agents (24, 25).

Ferumoxide, a suspension including iron oxide nanoparticles (IONs) with dextran coating, is used *in vivo*, admitted by FDA. These particles are degradable and metabolized by cells while entering to normal metabolic pathways. For example it increases serum iron level in one day and ferritin level in 7 days. However, ferumoxide has negative charge that without changing the surface charge of its particles is unable to connect the cells (26, 27). In this research, cell labeling was started on Hela cells as a model that was followed by labeling ESCs with different concentrations of ferumoxide-protamine sulfate complex. The best outcome belonged to the concentration of 100 μg ferumoxide and 3 μg protamin sulfates per each culture media in 24 hours for ESCs. Our results showed that companionship of transfection agents with ferumoxide can increase efficiency of cell labeling which confirmed the result published by Arbab et al. (28). According to Au et al. (29), after labeling mesenchymal stem cells (MSCs) with IONs, the percentage of cell viability and proliferation didn’t changed, whereas apoptosis increased in 3 days after labeling. In this study, the growth rate and viability of labeled and unlabeled mESCs in undifferentiated state were evaluated for first time. The results demonstrated that the growth of mESCs after 48 and 96 hours didn’t have a significant difference and IONs didn’t affect the growth, proliferation and viability of mESCs (P>0.05). As indicated by Partlow et al. (30), labeling bone marrow (BM)-MSCs with IONs in natural condition doesn’t alter the viability and proliferation of those cells. Some other types of covered IONs have been successfully used to label mammalian cells. Delcroix et al. (31) indicated that SPIONs covered with 1-hydroxyethylidene-1.1-bisphosphonic acid (HEDP) could label rat MSCs without any significant side effect on viability and differentiation ability.

To investigate the possible changes in cell surface markers of mESCs, SPION-labeled surface markers of these cells were analyzed by flowcytometry. SSEA1expressed on mESCs in undifferentiated state is the most important surface marker that discriminates these cells from hESCs. CD117 is another surface marker that is expressed on undifferentiated ESCs (4). The result revealed no significant difference between labeled and unlabeled cell population for stemness surface marker SSEA1 and CD117. In parallel to show undifferentiated condition of mESCs, evaluation of CD34 and CD38 markers demonstrated no difference between groups. Therefore, it’s assumed that frumoxide-protamine sulfate complex doesn’t affect mESCs differentiation status. EBs containing SPIONs were cultivated in hematopoietic inducing medium containing SCF, IL3, IL6, Flt3 and TPO for a week. The expression levels of CD34 and CD133 expressing cell population in labeled cells as compared to unlabeled cells showed no significant difference, so it can be concluded that labeling cells with SPIONs along with protamine sulfate has no significant effect on hematopoietic differentiation (P>0.05).

Krejci et al. (3) showed that magnetic labeling of mESCs under standard conditions has undetectable effects on their self-renewal. Typical properties of mESCs, such as the high level of the transcription factor Oct-3/4 or presence of the membrane antigen SSEA-1, were stable during cultivation for 10 passages in undifferentiated conditions in the presence of two types of tested standard size SPIONs. Also, no apoptosis mESCs were detected. However, when the mESCs were committed to differentiation, the presence of SPIONs in cells modified these processes. It is probably a response to double stresses, differentiation and presence of SPION.

## Conclusion

According to the results of the present study, SPIONs-labeling method as MRI agents in mESCs has no negative effects on growth, morphology, viability, proliferation and differentiation that can be monitored *in vivo*, noninvasively. Non-invasive cell tracking methods are considered as new perspectives in cell therapy for clinical use and as an easy method for evaluating the placement of stem cells after transplantation.
